# Feast for thought: A comprehensive review of food allergy 2021-2023

**DOI:** 10.1016/j.jaci.2023.11.918

**Published:** 2024-03

**Authors:** Irene Bartha, Noorah Almulhem, Alexandra F. Santos

**Affiliations:** a Children’s Allergy Service, Evelina London Children’s Hospital, Guy’s and St Thomas’ Hospital, London, United Kingdom; b Department of Women and Children’s Health (Pediatric Allergy), School of Life Course Sciences, Faculty of Life Sciences and Medicine, School of Immunology and Microbial Sciences King’s College London, London, United Kingdom; c Department of Otolaryngology Head and Neck Surgery, King Fahad Hospital of the University, College of Medicine, Imam Abdulrahman Bin Faisal University, Dammam, Saudi Arabia; d Peter Gorer Department of Immunobiology, School of Immunology and Microbial Sciences King’s College London, London, United Kingdom

**Keywords:** Food allergy, diagnosis, severity, immunotherapy, omalizumab, IgE, skin prick test, basophil activation test

## Abstract

A review of the latest publications in food allergy over the past couple of years confirmed that food allergy is a major public health concern, affecting about 8% of children and 10% of adults in developed countries. The prevalence of food allergy varies around the world, with the increase being driven mainly by environmental factors, possibly together with genetic susceptibility to environmental changes. A precise diagnosis of food allergy is extremely important. Both new tests (eg, the basophil activation test) and improved optimization of information provided by existing tests (eg, the skin prick test and measurement of specific IgE level) can contribute to improving the accuracy and patients’ comfort of food allergy diagnosis. Understanding the underlying immune mechanisms is fundamental to designing allergen-specific treatments that can be safe and effective in the long term. New discoveries of the immune response to food allergens, including T-cell and B-cell responses, have emerged. Novel therapeutic approaches are being trialed at various stages of development as attempts to allow for more active intervention to treat food allergy. Prevention is key to reducing the increase in prevalence. Early introduction of allergenic foods seems to be the most effective intervention, but others are being studied, and will, it is hoped, lead to modification of the epidemiologic trajectory of food allergy over time.

Food allergy (FA) is a major public health issue globally, affecting about 8% of children and 10% of adults (see Fig 1^1^, ^2^, ^3^, ^4^, ^5^, ^6^, ^7^, ^8^, ^9^, ^10^, ^11^, ^12^, ^13^, ^14^, ^15^, ^16^, ^17^, ^18^, ^19^, ^20^, ^21^, ^22^, ^23^, ^24^, ^25^, ^26^, ^27^, ^28^, ^29^, ^30^, ^31^, ^32^, ^33^, ^34^, ^35^, ^36^, ^37^, ^38^, ^39^, ^40^, ^41^, ^42^, ^43^, ^44^, ^45^, ^46^, ^47^ for global prevalence data). FA has a negative impact on the lives of patients with allergy and their families, and there is no curative treatment.^1^^,^^2^ Improved understanding of the genetic basis and immune mechanisms of this condition is important to finding target therapies that can modify the current epidemiologic context. Recently, there have been new developments in research and clinical guidelines in the field that researchers, clinicians and the public should be aware of. This review highlights the new evidence published over the past couple of years on the epidemiology, genetics, immune mechanisms, diagnosis, treatment, and prevention of FA.

**Fig 1:**
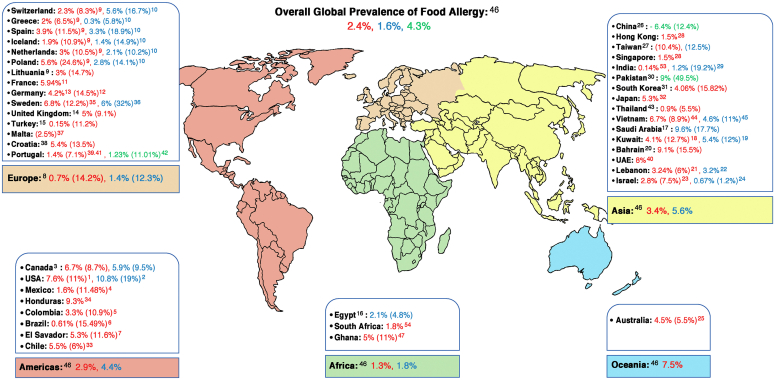
Prevalence of FA around the world according to clinical diagnosis of FA (represented in bold) and self-reported FA (indicated between parentheses). Clinical diagnosis of FA is defined as physician-confirmed FA based on suggestive history and allergen-specific IgE evidenced by skin prick testing and/or measurement of specific IgE, or based on challenge-proven FA. Different age groups are represented in different colors (*adults in blue;children inred*; *and all age groups, including adolescents,in green*).

## FA epidemiology and genetics

Hospital admissions for food-induced anaphylaxis have increased over recent decades; fortunately, however, fatalities have not increased.^48^^,^^49^ According to a recent UK study, the most affected age groups are children and teenagers and the most common causes of food-induced anaphylactic deaths are cow’s milk (CM) and nuts.^49^

The prevalence of FA varies in different areas around the world, being more common in developed countries and urban areas (Fig 1). A recent systematic review (SR) estimated a pooled lifetime prevalence and point prevalence of self-reported FA in Europe to be 19.9% and 13.1%, respectively.^8^ Point prevalence of FA based on specific IgE (slgE) level was 16.6% as opposed to 5.7% based on a skin prick test, and it was only 0.8% based on oral food challenge (OFC).^8^ Another recent SR and meta-analysis compared estimates of the 8 most common FAs in Europe between the periods 2000-2012 and 2012-2021 and concluded that the prevalence of allergy to these foods has not changed between the 2 time periods.^50^

FA prevalence can be affected by age at diagnosis and the type of FA, as certain FAs are more likely to be outgrown than others. In the HealthNuts study, the prevalence of peanut allergy (PA) was similar at ages 1 and 6 years (3.1%), whereas egg allergy (EA) had a prevalence of 9% at age 1 year, decreasing markedly to 1.2% by age 6 years. Even though 29% of cases of PA resolved, new onset of PA beyond 1 year was observed to occur at a rate of 0.7%, whereas no such new onset was observed for EA.^51^ Interestingly, FA prevalence among Australian children of Asian origin was 15% and that among Singaporean children was 1.1%,^52^ which suggests a possible role of ethnicity, environmental factors, and change in environment in the development of FA, particularly in the early years of life.

An accurate estimation of FA prevalence in developing countries is difficult on account of sparse data. A multicenter epidemiologic survey done as part of the EuroPrevall study, conducted in China and India, showed a wide variability in FA prevalence between the 2 countries (1.50% in Hong Kong, 0.21% in Guangzhou, 0.69% in rural Shaoguan, and 0.14% in India) that could not be explained by the degree of urbanization.^53^ In contrast, in the South African Food study (SAFFA), different estimates of FA were found between urban Capetown (2.5%) and rural areas (0.5%).^54^

In Southern Europe, nonspecific lipid transfer protein (nsLTP) allergy is considered the most common cause of primary FA and food-induced anaphylaxis.^55^ However, nsLTP allergy has been emerging in other countries in Northern Europe, China, Japan, and Latin America. Cannabis was recently identified as a primary sensitizer to nsLTP (namely *Cannabis sativa* 3) via direct contact or passive inhalation.^56^

Family history of FA and atopic diseases is a recognized risk factor for FA. Specific human leukocyte antigen (HLA) polymorphisms have been associated with PA. For instance, in the Learning Early about Peanut Allergy (LEAP) study, an association between peanut-specific IgG4 and *HLA-DQA1∗01:02* was observed in the peanut consumption group across the 38 alleles tested. This association, driven by IgG4 specific for *Arapis hypogaea* (Ara h) 2, had a positive correlation with peanut consumption.^57^ Notably, this confirmed the previously reported association of *HLA-DQA1∗01:02*, in addition to *MALT1* and its single-nucleotide variant rs57265082,^58^ with risk of PA development in the peanut avoidance group.^59^ Other HLA polymorphisms, namely, *HLA-DPB1∗02:01:02* and the single-nucleotide polymorphism rs9277630, were associated with increased risk of development of wheat-dependent exercise-induced anaphylaxis in a Japanese study.^60^

## Immune mechanisms of FA

**Fig 2:**
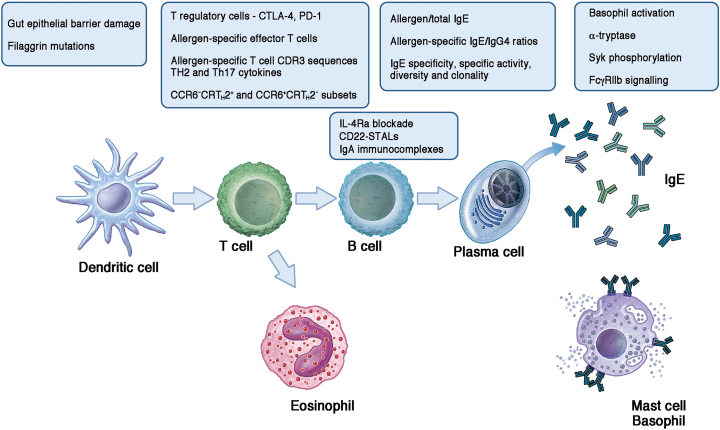
Highlights on the topics of the novel publications about mechanisms of FA and tolerance mentioned in the text.

Various lessons on the pathologic immune response to food allergens have emerged from mouse and *in vitro* studies of FA and from clinical trials of allergen-specific immunotherapy (AIT) during which blood samples were collected and assessed over time (Fig 2).

### Findings from animal studies

It is worth highlighting an interesting and somehow reversed study that tried to mimic a clinical trial, the LEAP study, in an animal model, using BALB/c mice fed commercial peanut butter in the presence of environmental peanut exposure.^61^ As expected, the mice that were fed peanut butter had less peanut sensitization and PA. This seemed to be mediated by human cytotoxic T lymphocyte–associated antigen-4 (CTLA-4) and the induction of regulatory T (Treg) cells. Another possible target for suppression of allergic responses to food is the programmed death 1 (PD-1)/programmed death ligand 1 (PD-L1) axis. Contrary to the evidence from autoimmune diseases and respiratory allergies, blocking the PD-1/PD-L1 interaction between T follicular helper cells and B cells suppressed high-affinity IgE responses and prevented anaphylaxis.^62^ In another model of PA, increased gut permeability led to anaphylaxis in response to peanut following oral exposure in sensitized mice, highlighting the importance of the gut epithelium and barrier integrity in the protection from developing FA.^63^ The importance of gut epithelial barrier was documented in a separate *in vitro* study in which dishwasher detergents and rinse aids were shown to be cytotoxic and damaging to the barrier.^64^ These studies support the hypothesis that damage to the epithelial barrier may be involved in the inception of FA and the increase in FA incidence over the past few decades.^65^^,^^66^

### Skin and mucosal barriers and allergens

The increased susceptibility to FA in subjects with filaggrin mutations due to facilitated allergen skin penetration into a proinflammatory milieu is well documented. Using samples from the German Genetics of Food Allergy Study (GOFA), Kalb et al confirmed this link and established, for the first time, an association between filaggrin loss-of-function mutations and the persistence of cow's milk allergy (CMA) and EA over time.^67^ The biochemical properties of food allergens also have an important role in allergen sensitization to foods. Chakrapani et al studied the allergenicity of alpha-gal in glycoproteins and glycolipids and found that alpha-gal present in both molecular complexes contributes to the allergic response, but a higher amount of allergen is present in the protein fraction.^68^ One of the allergenic foods that has commanded more attention and has accummulated more evidence over the years is peanut. Within peanut, Ara h 2 is considered the most important allergen in terms of its ability to cause effector cell activation and, consequently, allergic reactions. Interestingly, immunodominant conformational and linear IgE epitopes lie in a single segment of Ara h 2, as demonstrated by structural studies and by an independent study of a cohort of peanut-sensitized children tested initially on a peanut allergen microarray followed by validation of IgE binding measurements using the ImmunoCAP (Thermo Fisher Scientific, Waltham, Mass) platform.^68^, ^69^, ^70^, ^71^ A previous study published in the *Journal of Allergy and Clinical Immunology* reported immunologic cross-reactivity between the peanut major allergens Ara h 1, Ara h 2, and Ara h 3.^72^ Warmenhoven et al^73^ recently published evidence that this cross-inhibition between Ara h 1 or Ara h 3 and Ara h 2 was due to formation of complexes between the major allergens and contamination of natural Ara h 1 and Ara h 3 with 2S albumin Ara h 2. For this reason, recombinant allergens are preferred for diagnostics, as cross-contamination with Ara h 2 could lead to overestimation of the clinical relevance of Ara h 1 and Ara h 3 sensitizations.

### Allergen-specific T-cell responses

Single-cell transcriptomics, together with multiparameter flow cytometry, have shed light on T-cell responses to food allergens and their modulation by AIT.^74^ Ruiter et al^75^ identified biomarkers that were distinct in more sensitive patients with PA (ie, those reactive to ≤300 mg of peanut protein) than in patients with peanut allergy with a threshold higher than 300 mg of peanut protein (so-called hyporreactive patients). Such biomarkers include peanut-specific IgE levels, ratio of peanut to total IgE level (ie, specific activity), ratio of peanut-specific IgE level to IgG4 level (all higher in patients with PA), and importantly, higher frequency of peanut-specific effector T cells, with higher numbers of peanut-specific T-cell CDR3 sequences (both private and public) and greater production of T_H_2 and T_H_17 cytokines. Berin et al^76^ corroborated that peanut-specific T_H_2 cells were associated with a lower threshold of reactivity and correlated with allergen-specific IgE levels and basophil activation. Furthermore, this T_H_2 cell profile at baseline predicted an adverse response to AIT and decreased over the course of oral immunotherapy (OIT) to peanut and egg. CCR6^+^ peanut-specific T cells were inversely correlated with the T_H_2 cell population but were not directly related to clinical outcomes. Monian et al^77^ reported suppression of T_H_2 and T_H_1 effector cells (but not T follicular helper subsets) during OIT, and they associated a positive outcome with the T_H_2 cell suppression and treatment failure with preexisting T_H_1 and T_H_17 subsets that were not suppressed by OIT. More recently, Calise et al confirmed the existence of 2 mutually exclusive immunotypes of peanut-specific T cells defined by CCR6^−^CRT_H_2^+^ and CCR6^+^CRT_H_2^−^, and they described the immune trajectories for T-cell responses in children treated with peanut OIT in the IMPACT study, with clinical response being associated with the elimination of T_H_2A cells.^78^

### Allergen-specific B-cell responses

Exciting findings in B-cell biology in FA have been published over the past few years. An example is the finding that both central production of IgE in the bone marrow and IgE production in the gastrointestinal tract seem to be clinically relevant.^79^^,^^80^ To address the discrepancy between IgE sensitization and clinical FA, Hemmings et al studied functional characteristics of IgE and demonstrated that IgE specificity, specific activity, diversity, and clonality all independently influence mast cell (MC) and basophil activation following allergen stimulation. In the peanut IgE model, diversity and specific activity were the major drivers of basophil and MC activation. Focusing on epitope specificity, Suprun et al and Suarez-Farinas et al^81^^,^^82^ investigated the utility of peanut peptide–specific IgE to predict later development of PA and how this evolved over the course of the LEAP Study. They found that levels of peptide-specific IgE at age 3 to 15 months and age 2 to 3 years, together with peanut-specific IgE levels, predicted PA outcome after the age of 4. Interestingly, peanut consumption drove an increase in peptide-specific IgG4 early in all children, but this increase was more pronounced in peanut consumers, whereas peptide-specific IgE levels leading to PA (more common in peanut avoiders) increased significantly after age 2.5 years.

Trying to target the B-cell pathway to suppress allergen-specific antibody production, Bruton et al^83^ used a combination of *in vitro* and animal models to assess the effect of IL-4Rα blockade on peanut-specific recall responses. Although IL-4Rα blockade significantly abrogated the recall response and protected from anaphylaxis in the mouse model, in human *in vitro* studies it reduced IgE production and T_H_2 cytokine production, although the terminally differentiated T_H_2A population resisted independently of suppression of the pathway downstream the IL-4Rα. Another potential therapeutic approach for FA was attempted by Hardy et al^84^; their approach consisted of targeting CD22 on memory B cells with sialic acid–binding Ig-like lectin (SIGLEC)-engaging tolerance-inducing antigenic liposomes (STALs) codisplaying peanut allergens (Ara h 1, Ara h 2, or Ara h 3) and high-affinity CD22 ligand (CD22L-STALs). CD22L-STALs significantly suppressed systemic memory to all 3 major peanut allergens in both mouse models and in human B cells *in vitro*.

### Allergen-specific antibodies

The physiologic role of IgE-mediated hypersensitivity is the defense against parasitic infections, and this has generated interest in the comparison between antigens from parasites and those from allergenic sources. Hadadianpou et al^85^ were able to generate naturally occurring helminth-specific mAbs from filarial parasiste–infected subjects and demonstrate their ability to induce anaphylaxis in a passive sensitization mouse model. Similarities between parasitic antigens and allergens may help us to understand what makes an allergen an allergen and to predict allergenicity of new food sources coming into the food chain. Current understanding of the role of IgA in FA is limited. Zhang et al have shown that IgE and IgA production are the result of different immune pathways.^86^ Liu et al^87^ have tested many plasma and stool samples from a variety of well-characterized cohorts of food-allergic patients and nonatopic individuals and found that individuals with PA have higher levels of peanut-specific IgA than do individuals without PA. However, gut peanut-specific IgAs have different epitope specificities from those of plasma peanut-specific IgE and do not predict later development of PA or acquisition of tolerance. A similar observation was made for EA. These findings question the protective effect of mucosal IgA in FA. Surprisingly, using established mouse models, Elesela et al^88^ found that IgA immunocomplexes applied to gut mucosal surfaces reduced T_H_2 cell–driven immune responses, including reduced T_H_2 cytokine production and enhanced IL-10 and TGF-β production and increased Treg cell numbers, following allergen challenge. Interestingly, this regulatory response was antigen-specific and opened the way for potential new therapeutic strategies.

### Effector cells: MCs and basophils

Finally, with regard to effector cells of FA and anaphylaxis, a recent study associated the severity of food allergic reactions with α-tryptase.^89^ In an EA study, the basophil activation test (BAT) was the best immune marker to identify severe reactors.^90^ Interestingly, no demographic or clinical features and only 1 immune marker in addition to the BAT were statistically significantly different between patients with EA who experienced severe reactions and patients with EA who experienced mild or moderate reactions during baked egg challenges. Peanut OIT has been shown to induce basophil hyporesponsiveness, owing either to a decrease in IgE-mediated activation and/or to an increase in inhibitory signaling. In a small peanut OIT study, Kulis et al^91^ showed that the decrease in CD63 expression was accompanied by a decrease in Syk phosphorylation. Whether there is also a decrease in phosphorylation of regulatory signaling molecules, such as SHIP or SHP-2, with treatment remains to be seen. Kanagaratham et al^92^ used bone marrow–derived MCs from wild-type and FcγRIIβ-deficient mice to demonstrate that many IgE-mediated pathway genes were expressed following IgE receptor cross-linking, with a peak at 1 hour, and that antigen-specific IgG could modulate this by activating genes downstream the FcγRIIβ, ultimately leading to suppression of the Syk signaling pathway.

## FA diagnosis

OFC is the criterion standard for definitive diagnosis of FA. Recently, EAACI guidelines on the diagnosis of IgE-mediated FA recommend open OFC for most clinical situations, with double-blind placebo-controlled food challenge being reserved for cases in which open OFCs are equivocal and for research.^93^ The PRACTALL guidelines on how to perform OFC are widely used. The Consortium for Food Allergy Research, version 3 (CoFAR, v3), which uses less-objective symptoms and less-rigid challenge stop criteria, was recently proposed.^94^ This proposed approach can potentially compromise the reproducibility of the OFC and overestimate FA, and it did not seem to reduce the anaphylaxis rate much.^95^ Importantly, allergen thresholds can vary even in the absence of any intervention; Turner et al^96^ and Patel et al^97^ have recently shown that thresholds can vary within half a log (equivalent to 1 PRACTALL dosing interval) in peanut and CM allergic patients.

OFCs are time-consuming and costly, and they involve the risk of potentially severe allergic reactions. Thus, there has been a constant search for better diagnostic tools. A comprehensive SR and meta-analyses of studies on the utility of tests to support the diagnosis of FA, in which at least a proportion of patients underwent OFC, has recently generated useful indicative cut-offs with sensitivity and specificity that can be used to guide interpretation of allergy test results.^98^ Novel diagnostic *in vitro* tests with promising results include cellular tests, namely, the BAT and MC activation test (MAT), and serologic tests such as measurement of epitope-specific IgE level. These tests are promising future diagnostic tools for FA, with the BAT having an advantage over epitope-specific IgE testing for being a functional assay that combines all IgE characteristics.^99^

IgE to epitope-containing peptides reduces the possibility of cross-reactivity and has higher specificity than does IgE to whole allergen. It may, however, produce false-negative results owing to a smaller IgE-binding region and the possibility of missing the conformational epitopes, which play an important role in antibody binding. To minimize false-negative results, testing for specific IgE (sIgE) to allergen components can be done in addition.^99^^,^^100^ Using peptide microarray, testing for Ara h 2 peptide–specific IgE added diagnostic value to Ara h 2–specific IgE in diagnosis of PA, and the best was to consider IgE to Ara h 2 and to 4 of its peptides simultaneously.^70^ Of those 4 Ara h 2 peptides, 3 shared amino acid motifs, with sequences identified as immunodominant in separate studies done using the bead-based epitope assay (BBEA),^71^^,^^101^

In a subset from the LEAP study cohort, BBEA to test for IgE binding to 64 informative epitopes was used to compare peptide-sIgE and peptide-sIgG4 at baseline and again at 12, 30, and 60 months of age. The BBEA showed similar results at baseline in both the avoidance and consumption groups. However, peptide-sIgE expansion occurred mainly in the avoidance group, particularly in patients sensitized at baseline. Interestingly, peptide-sIgE expansion was observed primarily after 2.5 years. On the other hand, peptide-sIgG4 expansion appeared in all groups, but was found earlier in the consumer group (particularly in sensitized children) during the first 30 months.^82^ Using data from the same study, researchers performed BBEA on 74 patients from among the LEAP participants in combination with specific IgE of peanuts and its components to assess its ability to predict the outcome of OFC at age 5 years. An algorithm combining peptide-sIgE and IgE to Ara h 1, Ara h 2, Ara h 3, and Ara h 9 proteins gave 64% validation accuracy at baseline and 83% accuracy of samples taken at 1 year to predict the OFC outcome at 5 years.^102^

The BAT and the MAT are flow cytometry–based tests that involve all components of the allergic reaction, including effector cells, sIgE, and allergen-specific antibodies of other isotypes. When compared with sIgE assays, they provide a result that is closer to the patient’s allergic status. In a recent publication of the BAT2 study (National Clinical Trials [NCT] no. 03309488), the BAT proved to be the best diagnostic utility for EA from the standpoint of diagnostic accuracy.^103^ An approach of applying 100% sensitive and 100% specific cutoffs to select patients who are without allergy and tolerant, respectively, followed by additional tests or OFC in equivocal cases, was proposed and provided 100% diagnostic accuracy. The BAT provided the greatest reduction in OFC in all age groups when used as a single test. Using BAT as a second step after testing for sIgE to egg white or ovalbumin reduced the number of BATs required, providing a similar reduction in OFC use and 100% diagnostic accuracy, thus emphasizing its role as a second test to improve diagnostic accuracy. In the same study, the BAT was the best predictor of severity and threshold of allergic reaction, providing 76% sensitivity, 74% specificity, and 75% accuracy to identify severe reactors to baked egg. As for identification of patients who react to low doses of allergen, the BAT showed 70% sensitivity, 72% specificity, and 71% accuracy.^90^ In a PA study, the BAT demonstrated a high reproducibility and reliability when tested in different laboratories.^104^

Compared with the BAT, the MAT has shown similar specificity but lower sensitivity in the diagnosis of PA.^99^^,^^105^ However, in the 10% to 15% of individuals who have nonresponding basophils, the MAT can provide conclusive results.^100^^,^^105^ A proposed novel MAT assay uses conditional Hoxb8-immortalized progenitor-derived mouse MCs transgenic to human high-affinity IgE receptor (FcεRIa) passively sensitized with patient’s serum and analyzed by flow cytometry following allergen stimulation, as in the original MAT.^106^ Recently, the BAT was, for the first time, included as a recommended test to support the diagnosis of IgE-mediated FA in clinical guidelines.^93^ However, it was decided that the MAT and peptide-sIgE required more research before being used clinically.

Following a definitive diagnosis, delineating the severity of FA is important to identify patients who need a more intense follow-up and may have an indication for selective treatments. However, doing so can be difficult because the severity of allergic reactions is multifactorial and many factors depend on the circumstances in which the allergic reaction occurs, which are in turn unpredictable. Risk factors for severity have been defined from reviewing cohorts with fatal and near-fatal reactions. However, large longitudinal population-based studies are needed to be able to accurately risk-stratify patients with FA. In addition, patients might still develop severe reactions in the absence of risk factors.^107^ Nevertheless, having a way to more holistically categorize both severity of allergic reactions and disease severity in an objective and unified manner would be helpful. Fernández-Rivas et al developed the Food Allergy Severity Score (FASS) based on multidisciplinary expert consensus and mathematic modeling of data from the EuroPrevall study. Versions of the FASS include the oFASS-3 (which uses 3 grades [mild, moderate, and severe]), the oFASS-5 (which uses 5 grades, scored 1-5), and the nFASS (which uses a numeric scale). Its internal validation was followed by external validation using 5 cohorts.^108^ The Definition of Food Allergy Severity (DEFASE) scoring system was proposed following an international expert consensus using the Delphi method. The DEFASE consists of 5 domains, including signs and symptoms of the most severe reaction, the minimum therapy to treat the most severe reaction, individual eliciting dose, current FA-related quality of life, and economic impact of FA severity. Each domain is scored as mild (1 point), moderate (2 points), or severe (3 points), and the overall DEFASE score is the sum of the scores of all 5 domains.^109^ For a summary of the diagnostic performance of the reported optimal cutoffs for several of the aforementioned tests, see Table I.

**Table I:** Summary of the diagnostic performance of reported optimal cutoffs for different tests for specific food allergies based on recent meta-analyses of studies including children and/or adults

Diagnostic test		CM (allergy to fresh pasteurized CM)	EA (allergy to whole cooked egg)	PA	Hazelnut allergy	Cashew nut allergy	Sesame seed allergy	Wheat allergy	Shrimp allergy
SPT	Cutoffs (mm)	4 (3-8)	5 (3-8)	4 (3-8)	5 (3-7)	5 (4-6)	8 (4-10)	3 (3-5)	3 (3-5)
	Sensitivity	0.52 (0.24-0.79)	0.68 (0.37-0.88)	0.84 (0.69-0.92)	0.82 (0.68-0.91)	0.93 (0.89-0.96)	0.70 (0.55-0.82)	0.53 (0.23-0.81)	0.62 (0.44-0.77)
	Specificity	0.80 (0.65-0.90)	0.77 (0.64-0.86)	0.86 (0.79-0.91)	0.78 (0.44-0.94)	0.92 (0.82-0.96)	0.89 (0.76-0.95)	0.72 (0.57-0.84)	0.90 (0.31-0.99)
sIgE to allergen extracts	Cutoffs (KU/L)	3.5 (0.9-10.5)	3.5 (1.7-5.5)	4.3 (0.35-10)	2.34 (0.6-6.3)	1.1 (0.6-3.1)	7.5 (0.9-50)	0.6 (0.35-5.6)	1.2 (0.5-3.1)
	Sensitivity	0.82 (0.59-0.94)	0.85 (0.77-0.90)	0.81 (0.71-0.88)	0.79 (0.71-0.85)	0.94 (0.89-0.97)	0.70 (0.23-0.95)	0.72 (0.54-0.84)	0.96 (0.42-1.00)
	Specificity	0.92 (0.80-0.97)	0.73 (0.63-0.80)	0.83 (0.71-0.90)	0.62 (0.38-0.81)	0.64 (0.54-0.74)	0.83 (0.26-0.99)	0.79 (0.68-0.86)	0.63 (0.46-0.78)
sIgE to allergen components	Cutoffs (KU/L)	Casein 2.6 (1.0-5.3)	Ovomucoid 0.8 (0.35-3.7)	Ara h 2 0.44 (0.3-1.3)	Cor a 14 0.64 (0.35-3.5)	Ana o 3 0.4 (0.2-0.6)		Omega-5-gliadin 0.3 (0.1-0.6)	Pen a 1 1.1 (0.6-4.4)
	Sensitivity	0.67 (0.53-0.78)	0.74 (0.54-0.87)	0.82 (0.77-0.86)	0.73 (0.53-0.87)	0.96 (0.91-0.98)		0.79 (0.68-0.88)	0.62 (0.45-0.76)
	Specificity	0.93 (0.85-0.97)	0.91 (0.87-0.93)	0.92 (0.87-0.95)	0.95 (0.90-0.98)	0.94 (0.88-0.97)		0.78 (0.66-0.86)	0.89 (0.75-0.95)
BAT	Cutoffs (% CD63^+^ basophils)			5.0 (4.7-7.1)			10.9 (8.2-11.6)		
	Sensitivity			0.84 (0.76-0.90)			0.89 (0.80-0.94)		
	Specificity			0.90 (0.83-0.94)			0.93 (0.76-0.98)		

All ranges in parentheses are 95% CIs.

Data from Santos et al^93^ and Riggioni et al.^98^

*Ana o*, *Anacardium occidentale*; *Cor a*, *Corylus avellana*; *Pen a*, *Penaeus aztecus;SPT,* skin prick test*.*

## FA treatment

### Food allergen immunotherapy

AIT for FA has gained adepts, both among clinicians and among patients. Recent studies have addressed important remaining questions that concern clinicians, researchers, and patients alike; these concerns include the heterogeneity of study design, protocol, outcomes and risk of bias, the ideal patient profile, the best route of administration, duration of treatment, and associated management of FA in terms of food avoidance and need for epinephrine autoinjectors. Table II shows recent and ongoing studies in this area.

**Table II:** Current and ongoing research studies examining treatment of FA

Study name (NCT or ACTRN no.)	Study description	Estimated enrollment	Target age groups	Main Outcomes
**OIT**				
Using Commonly Available Food Products to Treat Food Allergy (NATASHA Study; NCT05503446)	Multicenter, randomized, parallel assignment, phase III of OIT to peanut or CM, with crossover for those allocated to control group after primary outcome (∼24 mo)	N = 216	3-23 y	Proportion of participants in intervention arms (combined) vs in control arm (by allergen) who, following 9-12 mo of OIT, tolerate ≥1 g of food protein (discrete dose, ∼4-6 peanuts or 30 mL of CM) *AND* demonstration of a minimum 10-fold increase in the MTD (defined as the highest dose not causing dose-limiting symptoms) at DBPCFC at the end of updosing vs at baseline DBPCFC
**Multifood OIT**				
ADP101 for Oral Immunotherapy in Food-Allergic Children and Adults (the Harmony Study; NCT04856865)	Phase I and II, multicenter, randomized, double-blind, placebo-controlled study (average of 1 y) of ADP101, an active powder formulation for OIT	N = 73	4-55 y	Proportion of subjects who tolerate a dose of ≥600 mg of protein of the relevant allergen or allergens with no more than mild symptoms at the exit DBPCFC
Open-label Extension Study of ADP101 (NCT05243719)	Phase I and II, open-label, safety extension study for participants who participated in the Harmony study (protocol ADP101-MA-01; ∼4-6 y)	N = 45	4-57 y	Long-term safety and tolerability of ADP101, including the incidence of adverse events during the study period
Low-Dose Multi-OIT for Food Allergy (LoMo) (LoMO Study; NCT03799328)	Phase II, single-center, single-arm, open-label study (∼18 mo) of multifood OIT	N = 18	6 mo-15 y	Change in MTD in a dichotomous manner and immunologic change in IgG4 level over 18 mo
**EPIT**				
Follow-up of the EPITOPE Study to Evaluate Long-term Efficacy and Safety of DBV712 in Young Children (EPOPEX Study; NCT03859700)	Phase III, multicenter, open-label, follow-up study for subjects who completed the EPITOPE study (24-36 mo)	N = 330	2-5 y	Proportion of subjects reaching an ED of ≥1000 mg at ages 12, 24, and 36 mo
Safety and Efficacy Study of Viaskin Peanut in Peanut-Allergic Children 4-7 Years of Age (VITESSE Study; NCT05741476)	Phase III, multicenter, randomized, double-blind and placebo-controlled study (∼58 wk) of daily DBV712 250 μg	N = 600	4-7 years	Proportion of responders in active treatment vs in the placebo group. A participant is defined as a treatment responder if the initial ED was ≤30 mg of peanut protein and the ED was ≥300 mg of peanut protein at the posttreatment DBPCFC at mo 12 *OR* the initial ED was > 30 mg of peanut protein and the ED is ≥600 mg of peanut protein at the posttreatment DBPCFC at mo 12
Efficacy and Safety of Viaskin Milk in Children with IgE-Mediated Cow's Milk Allergy (MILES Study; NCT02223182)	Phase I and II, multicenter, randomized, double-blind and placebo-controlled study (12 mo)	N = 198	2 to 17	Proportion of subjects who are treatment responders after 12 mo of EPIT treatment. Treatment responder is defined as a subject who meets ≥1 of the following criteria: • A ≥10-fold increase in the CRD of CM proteins at the mo 12 DBPCFC vs value at baseline and reaching ≥144 mg of CM proteins; • A CRD of CM proteins of ≥1444 mg at the 12-mo DBPCFC
**Oralmucosalimmunotherapy**				
OMEGA Study: A Study of the Safety and Feasibility of Uptitration with INT301 in Adults with Sensitivity to Peanut (the OMEGA Study; NCT04603300)	Phase I, multicenter, randomized, double-blind, placebo-controlled study (48 wk) of INT301, a fully functional peanut toothpaste containing immunotherapy agents able to deliver ≥50 mg of peanut product per dose vs 2-3.7 mg per dose in SLIT	N = 32	18-55 y	• Proportion of participants able to consistently tolerate the protocol-specified highest dose • Incidence of systemic and nonsystemic adverse reactions will be evaluated
**SCIT**				
HAL-MPE1 Safety and Tolerability Study (NCT02991885)	Phase I, multicenter, randomized, double-blind, placebo-controlled study (an average of 16 wk) of HAL-MPE1, an off-white to white liquid suspension containing modified peanut extract delivered as SCIT	N = 42	5-50 y	Local and systemic reactions (immediate [≤1 h]), early [within 1-4 h], and late [>4 h]) and the occurrence of treatment-emergent adverse events To confirm its safety and tolerability of HAL-MPE1 in adults with PA and subsequently assess its safety and tolerability in adolescents and children with PA
**Combination of immunotherapies**				
Improving the Safety of Oral Immunotherapy for Cow’s Milk Allergy (SOCMA Study; NCT02216175)	Phase II and III, a 2-center, parallel group, 3-arm, randomized placebo- controlled trial (∼15 mo) of CM SLIT + OIT vs OIT 3 study arms: - SLIT followed by conventional OIT - Conventional OIT - Placebo followed by conventional OIT	N = 68	6-17 y	Proportion of participants experiencing adverse events (excluding mild, nontransient symptoms) conventional OIT for CM in phase 2, in those who have received SLIT before treatment vs placebo
**mAbs**				
Study to Evaluate Dupilumab Monotherapy in Pediatric Patients with Peanut Allergy (NCT03793608)	Phase II, multicenter, open-label, single-group assignment study (∼36 wk) of dupilumab as monotherapy in children with PA	N = 25	6-17 y	Proportion of participants treated with dupilumab who tolerate ≥444 mg (cumulative) of peanut protein during DBPCFC at wk 24
Study in Pediatric Subjects with Peanut Allergy to Evaluate Efficacy and Safety of Dupilumab as Adjunct to AR101 (Peanut Oral Immunotherapy; NCT03682770)	Phase 2, multicenter, randomized, double-blind, placebo-controlled study (∼76 wk) of dupilumab with peanut OIT in children with PA	N = 149	6-17 y	Proportion of participants treated with dupilumab + AR101 vs placebo + AR101 who tolerate 2044 mg (cumulative) of peanut protein in DBPCFC (time frame: ≤40 wk)
Dupilumab and Milk OIT for the Treatment of Cow’s Milk Allergy (NCT04148352)	Phase II, multicenter, randomized, double-blind, parallel group, 2-arm study (44 wk) of dupilumab with milk OIT in children and adults with CMA	N = 40	4-50 y	Proportion of subjects treated with dupilumab + milk protein OIT vs placebo + milk protein OIT who tolerate ≥2040 mg (cumulative) of CM protein during DBPCFC to milk at wk 18
Clinical Study Using Biologics to Improve Multi-OIT Outcomes (COMBINE Study; NCT03679676)	Phase II, multicenter, randomized, double-blind multiallergen OIT study of combination of biologics (omalizumab and dupilumab) and multifood OIT (∼44 wk) Food allergens evaluated include CM, almond, shellfish, fish, cashew, hazelnut, egg, walnut, sesame seeds, soy, and wheat	N = 110	4-55 y	Proportion of subjects able to tolerate peanut, peanut and ≥1 other food allergen, or peanut and 2 other food allergens at 44 wk
Omalizumab as Monotherapy and as Adjunct Therapy to Multiallergen OIT in Food-Allergic Participants (OUtMATCH Study; NCT03881696)	Phase III, multicenter, randomized, double-blind, placebo-controlled study (∼56 mo) of omaliumab as monotherapy and omalizumab with multifood OIT Food allergens evaluated include milk, egg, wheat, cashew, hazelnut, and walnut	N = 471	1-55 y	• Proportion of participants who successfully consume a single dose of ≥600 mg of peanut protein without dose-limiting symptoms during the DBPCFC at the end of stage I of treatment • Proportion of participants who consume foods without dose-limiting symptoms during a DBPCFC after treatment with either omalizumab or placebo for omalizumab
Omalizumab to Accelerate a Symptom-driven Multi-food OIT (BOOM Study; NCT04045301)	Phase IIb, multicenter randomized double-blind and placebo-controlled study (15 mo) of omalizumab at decreasing times to maintenance during a symptom-driven multifood OIT Food allergens evaluated include peanut, milk, egg, wheat, oat, soy, barley, rye, buckwheat, hazelnut, pecan, cashew, pistachio, almond, walnut, and sesame	N = 90	6-25 y	Time from initial food escalation to target multifood protein maintenance dose of 1500 mg of total food protein assessed ≤52 wk after initial food escalation
E-B-FAHF-2, Multi-OIT and Omalizumab for Food Allergy (NCT02879006)	Phase II, multicenter randomized double-blind and placebo-controlled study (∼26 mo) of Chinese herbal formula, multifood OIT, and omalizumab	N = 33	6-40 y	To evaluate sustained unresponsiveness by the absence of dose-limiting symptoms to a cumulative dose of 4444 mg of food allergen protein at mo 29 Food allergens evaluated include milk, egg, peanut, almond, cashew, hazelnut, walnut, sesame, and/or wheat
Efficacy and Safety of QGE031 (Ligelizumab) in Patients with Peanut Allergy (NCT04984876)	Phase III multicenter, randomized, double-blind, placebo-controlled study (52-wk) of 2 regimens for dosing ligelizumab (240 mg and 120 mg) via subcutaneous injection every 4 wk in participants with a medically confirmed diagnosis of IgE-mediated PA	N = 486	6-55 y	Proportion of participants who can tolerate a single dose of ≥ 600 mg (1044 mg cumulative tolerated dose) of peanut protein without dose-limiting symptoms at wk 12 Responder status is defined as tolerating a single dose of ≥ 600 mg (1044 mg cumulative tolerated dose) of peanut protein without dose-limiting symptoms during the DBPCFC conducted at wk 12
JAK Inhibition in Food Allergy (NCT05069831)	Phase I single-center, randomized, double-blind pilot study (4 mo) of abrocitinib Food allergens evaluated include peanut, cashew, walnut, hazelnut, sesame, cod, and/or shrimp	N = 40	18-50 y	Change in basophil activation and in SPT result after 4 mo of treatment vs at baseline
Adjuvant Treatment with Abatacept to Promote Remission during Peanut Oral Immunotherapy (ATARI Study, NCT04872218)	Phase IIa, multicenter, randomized, double-blind placebo-controlled trial of 24 wk of abatacept, a fusion protein of the extracellular domain of the CTLA-4 linked to the Fc domain of human immunoglobulin, vs placebo used as an adjuvant to OIT (∼48 wk overall)	N = 14	14-50 y	Change in peanut-specific/total IgE at wk 24 vs at baseline
Study of Efficacy, Safety and Tolerability of Remibrutinib in Adult Participants with an Allergy to Peanuts (NCT05432388)	Phase II, multicenter, randomized, investigator- and participant-blinded, placebo-controlled study of oral remibrutinib (LOU064) in 3 doses of oral tablet twice a day compared with placebo (∼1 mo or up to 5 wk)	N = 110	18-55 y	Proportion of participants who can tolerate a single dose of ≥600 mg of peanut protein without dose-limiting symptoms during DBPCFC at baseline and d 26 Responder status defined as tolerating a single dose of ≥600 mg of peanut protein without dose-limiting symptoms during the DBPCFC
**Microbiome-modulating agents**				
A Randomized, Controlled Trial of Probiotic and Peanut Oral Immunotherapy in Inducing Tolerance in Hong Kong Children with Peanut Allergy Compared with Oral Immunotherapy (OIT) Alone and with Placebo (NCT05165329)	3-Arm, randomized (4:4:1), stratified (by age), blinded, placebo-controlled, parallel-group, superiority trial of probiotic and peanut OIT (∼130 wk)	N = 90	1-17 y	Proportion of participants with an 8-wk sustained unresponsiveness (passed T1 and T2 challenges) in probiotic and peanut OIT vs placebo (T2 = 8 wk after final day of maintenance treatment) T1, 18 mo; T2, 8 wk; and T3, 12 mo
Probiotic Egg Allergen Oral Immunotherapy for Treatment of Egg Allergy: The PEAT Study	Phase II, dual-center, randomized, double-blind placebo-controlled study of egg OIT (∼21 mo)	N = 80	5-30 y	Proportion of participants who attain an 8-wk sustained unresponsiveness (remission) (passed T1 and T2 challenges) in active and placebo-treated groups T1, 18 mo from baseline; T2, 20 mo from baseline
Pinpoint Trial: Prebiotics in Peanut Oral Immunotherapy (NCT05138757)	Phase I and II, randomized, single-group assignment double-blind placebo-controlled study of prebiotics (dietary fiber) and peanut OIT (4 y)	N = 30	4-17 y	• Proportion of subjects who tolerate ≥1043 mg (cumulative) of peanut protein with no more than mild symptoms at the 12-mo DBPCFC (time frame: ≤4 y) • Reduction of side effects of OIT • Changes in the gut microbiota
Oral Peanut Immunotherapy with a Modified Dietary Starch Adjuvant for Treatment of Peanut Allergy in Children Aged 10-16 years (ACTRN12617000914369)	Randomized, blinded, parallel group, 3-arm, placebo-controlled trial of dietary fiber supplement that is high in a key short-chain fatty acid (butyrate) in combination with peanut OIT (∼25.5 mo) Study arm A: peanut OIT and a supplement control group (receiving LAMS, a low-amylose maize) Study arm B: peanut OIT and a supplement group (HAMSB, a butyrylated high-amylase maize starch) Study arm C: peanut OIT control (peanut avoidance)	N = 65	10-16 y	Proportion of participants who tolerate ≥1400 mg of roasted peanut at a DBPCFC after 12 mo of treatment followed by 6 wk of interruption of treatment
Evaluating the Safety and Efficacy of Oral Encapsulated Fecal Microbiota Transplant in Peanut Allergic Patients (NCT02960074)	Phase I, nonrandomized, single-group assignment, treatment study of oral encapsulated FMT in PA (∼1 y)	N = 15	18-40 y	Presence of FMT-related adverse events grade ≥2 (time frame: 12 mo)
Evaluating the Safety and Efficacy of Oral Encapsulated Microbiota Transplantation Therapy in Peanut Allergic Patients (NCT05695261)	Phase II randomized, double-blind, placebo-controlled study of oral encapsulated fecal MTT with antibiotic pretreatment	N = 24	12-17 y	Change in threshold of peanut reactivity during a DBPCFC from ≤100 mg of peanut protein to 300 mg after 28 days of MTT vs placebo therapy and 4 mo after therapy initiation
VE416 for Treatment of Food Allergy (NCT03936998)	Phase I/II, single-center, randomized, double-blind placebo-controlled study of oral encapsulated fecal MTT with antibiotic pretreatment (∼54 wk) VE416 is a consortium of commensal, or "friendly", dormant (inactive) bacteria given in a capsule. The bacteria are reactivated once they reach participants’ intestines Study arms: - Combination Product: vancomycin + VE416 before peanut OIT - Combination Product: vancomycin + VE416 with peanut OIT - Combination product: placebo + VE416 with peanut OIT - Combination product: placebo + placebo with peanut OIT	N = 60	12-55 y	Primary end point for phase Ib: no. of participants with treatment-related adverse events, as assessed by CTCAE, v4.0, by 7 wk Primary end point for phase II: geometric mean of the MTD of peanut protein at DBPCFC at 23 wk
**Other molecules**				
Phase I Trial to Evaluate VLP Peanut in Healthy and Peanut Allergic Subjects (PROTECT Study, NCT05476497)	Phase I, randomized, sequential assignment, interventional, 2-part clinical trial of SCIT to Ara h 2 using peanut VLPs (∼34 wk) 2-part clinical trial: Part A: open-label with 2 groups (A1 and A2): A1 (healthy subjects): subcutaneous (SC) dosing with ascending concentrations of peanut VLPs A2 (subjects with PA): SPT performed with ascending concentrations of peanut VLPs. Part B: double-blind, placebo-controlled part that will enroll subjects with PA: subcutaneous dosing with ascending concentrations of peanut VLPs	N = 58	18-50 y	No. and severity of adverse events, including local and systemic (time frame: group A1, 18 wk; part B, 64 wk (part B); group A2, 3 d) No. of subjects discontinuing prematurely from treatment owing to adverse events (time frame: group A1, 11 wk; part B, 15 wk)
A Safety and Efficacy Study of PVX108 in Children and Adolescents with Peanut Allergy (NCT05621317)	Phase II, multicenter, randomized, double-blind placebo-controlled study of PVX108 immunotherapy (∼74 wk)	N = 90	4-17 y	• Ratio of MTD of peanut protein at the wk 46 DBPCFC relative to baseline in children aged 4-11 y treated with PVX108 vs placebo
A Study to Evaluate Safety, Tolerability and Immune Response in Adolescents Allergic to Peanut after Receiving Intradermal Administration of ASP0892 (ARA-LAMP-vax), a Single Multivalent Peanut (Ara h1, h2, h3) Lysosomal Associated Membrane Protein DNA Plasmid Vaccine (NCT03755713)	Phase 1, randomized, parallel assignment, double blind, placebo-controlled study of intradermal administration of ASP0892 (∼576 d [18 mo and 28 d]) • ASP0892 (ARA-LAMP-vax): a single multivalent peanut (Ara h1, h2, h3) lysosomal-associated membrane protein DNA plasmid vaccine applied by intradermal administration Cohort A: experimental ASP0892, low dose Cohort B: experimental ASP0892, high dose Placebo comparator: placebo	N = 20	12-17 y	• Treatment-emergent adverse events (≤d 576) • Local reactogenicity reactions (≤d 50). Systemic reactogenicity reactions (≤d 50) anti–LAMP-1 antibody formation (≤d 576) • No. of participants with vital signs abnormalities and/or with laboratory value abnormalities and/or adverse events (≤d 576)
CNP-201 in Subjects with Peanut Allergy (NCT05250856)	Phase I and II, multicenter, randomized, quadruple blind, multiple ascending dose study of CNP-201: a comprised purified peanut extract substance dispersed within a special matrix. (∼67 d)	N = 58	16-55 y	Safe and tolerable dose level determined by evaluating the frequency of adverse events and serious adverse events (through study completion, an average of 67 d) and serum cytokine levels (an average of 52 d)

Details of the studies, including recruitment status, are as of October 2023 (https://clinicaltrials.gov/).

*ACTRN*, Australian New Zealand Clinical Trials Registry number; *CRD*, cumulative reactive dose; *CTCAE*, Common Terminology Criteria for Adverse Events; *DBPCFC*, double-blind placebo-controlled food challenge; *ED*, Eliciting dose; *FMT*, fecal microbiota transplantation; *JAK*, Janus kinase; *MTD*, maximum tolerated dose; *MTT*, microbial transplantation therapy; *NCT*, National Clinical Trial; *SCIT*, subcutaneous immunotherapy; *SPT*, skin prick test.

Baked milk OIT showed desensitization and a good safety profile after 12 months of treatment in children aged 3 to 18 years, with improvement in proxy-reported FA quality of life.^110^ OIT is more effective in preschool children, with higher rates of sustained unresponsiveness, desensitization, and even ad lib consumption following treatment, although whether these effects are long term is still unknown.^78^^,^^111^ Interestingly, in children with PA, remission was associated with both younger age and lower baseline peanut-sIgE levels.^78^ The superior efficacy could be due to the greater plasticity of the immune response in the preschool years; better adherence to treatment thanks to greater parental and caregiver supervision; milder phenotypes of PA; and lower likelihood of aversion, dislike, and/or anxiety around food.^111^

Epicutaneous immunotherapy (EPIT) has a good safety profile. The peanut patch VP250, which contains a dose of 250 μg of peanut protein, was safe and well tolerated in children with PA.^112^ Subjects with atopic dermatitis at baseline showed a higher trend of responder rates but also higher rates of local reactions.^113^ The desensitization effects of EPIT to peanut have improved with longer duration of treatments,^114^ so follow-up studies are currently ongoing to evaluate this in the long term. Other EPIT studies, namely, examining the effects of EPIT to CM, are ongoing.

Sublingual immunotherapy (SLIT) has shown an intermediate safety profile between OIT and EPIT, likely related to the dose of allergen administered and possibly to the route of administration. SLIT has been studied for PA and compared with OIT. Younger age groups and longer duration of SLIT were associated with higher dose of peanut protein tolerated; hence, as is the case with OIT, implementing this therapy earlier in life may be beneficial. Recently, an open-label prospective study in children with PA who were aged 1 to 11 years showed that peanut SLIT induced desensitization in 70% of the participants and 36% successfully consumed a dose of 5000 mg of peanut protein.^115^ Combination of SLIT and OIT is currently being trialed for CMA in the Improving the Safety of Oral Immunotherapy for Cow's Milk Allergy (SOCMA) study.

A new delivery platform for AIT called oral mucosal immunotherapy has been developed; it enables the delivery of higher allergen doses (compared with those used in SLIT) to areas in the oral mucosa with higher concentrations of Langerhans cells. Oral mucosal immunotherapy aims to improve safety, adherence, and efficacy versus with SLIT and is being tested in a phase I study of INT301 in adults with PA (the OMEGA study).^116^ SCIT is a route of administration that is also currently being trialed (eg, for treatment of PA).

### Biologics

An SR of the literature with meta-analyses has compiled evidence on the use of omalizumab to treat FA.^117^ Most such studies focus on the use of omalizumab as an adjuvant of OIT, with fewer studies assessing its efficacy as monotherapy. The SR concluded that omalizumab increased the dose of food tolerated compared to before treatment, reduced allergic reactions, and improved quality of life. As an adjunct of OIT, omalizumab facilitated dose escalation and higher maintenance doses. The results of the Outmatch study, the primary outcome of which is the efficacy of omalizumab as monotherapy for multiple FAs, are eagerly awaited. The safety and efficacy of pretreatment with omalizumab to use a lower maintenance dose in a multifood OIT regimen have been evaluated recently. The study compared 2 dose regimens, 300 and 1200 mg of total protein, including total doses of at least 2 and up to a maximum of 5 foods. On both study arms, 70% of the participants showed changes in the sIgG4/IgE ratio of at least 2 allergens, and no differences were found in terms of adverse events.^118^ Dosing studies of omalizumab to accelerate multifood OIT, either on its own (eg, the BOOM study) or together with E-B-FAHF-2 Chinese herbal therapy, are ongoing.

Another anti-IgE mAb, ligelizumab, which has an approximately 88-fold higher affinity for human IgE than omalizumab, is currently being trialed for PA in phase III multicenter study (NCT04984876). Dupilumab, a mAb against the α-chain of the IL-4 receptor inhibiting the signal for both IL-4 and IL-13, is being studied in combination with peanut OIT (NCT03793608 and NCT03682770), milk OIT (NCT04148352), and omalizumab and multifood OIT (NCT03679676).

Other pharmacologic agents being trialed in FA include the following: abrocitinib, an oral Janus kinase (JAK) inhibitor that is being tested in a phase I study for peanut, cashew, walnut, hazelnut, sesame, cod, and/or shrimp allergies in patients aged 18 to 50 years (NCT05069831); abatacept, a fusion protein of the extracellular domain of the human CTLA-4 that links to the fragment crystallizable (Fc) domain of human immunoglobulin and is being tested as an adjuvant to OIT in a phase II study of adolescents and adults with PA (NCT04872218); and remibrutinib, a Bruton tyrosine kinase (BTK) inhibitor that is being tested in a phase II study of adults with PA (NCT05432388). Acalabrutinib, another BTK inhibitor, has been shown to increase patients’ threshold dose of peanut protein in adults with PA.^119^

### Microbiome-modulating agents

Gut microbial composition, diversity, and metabolic activity are factors that can influence the balance between allergy and tolerance to certain foods. For instance, Chun et al^120^ followed high-risk infants with no PA up until about 9 years of age and found that 28.7% of the children developed PA over time. Children with PA had lower gut microbiome diversity and different abundances of *Clostridium* and *Bifidobacterium* species, with butyrate and isovalerate decreasing and histidine metabolites, like histamine, increasing over time.

In terms of clinical trials of probiotic supplementation, *Bifidobacterium bifidum* TMC3115 improved safety outcomes in children with CMA,^121^ and in another study,^122^ *Lactobacillus rhamnosus* GG facilitated recovery from gastrointestinal symptoms and improved fecal occult blood in children with CMA.^122^ Probiotics may improve the safety of AIT when given as an adjunct treatment. *Lactobacillus rhamnosus* GG ATCC 53103 reduced systemic reactions, gastrointestinal symptoms, and moderate-to-severe adverse events but did not affect the efficacy of peanut OIT in children, particularly those aged 1 to 5 years.^123^ A similar study in a Chinese population is currently ongoing (NCT05165329). However, neither study included a probiotic-only arm, thus preventing a proper measure of effect. Probiotics are also currently being studied in combination with egg OIT.^123^

Prebiotics (eg, dietary fiber [NCT05138757] or modified dietary starch [Australian and New Zealand Clinical Trial Registry number (ACTRN) 12617000914369]) are being evaluated as adjuvants of peanut OIT. The combination of synbiotics (prebiotics oligosaccharides and probiotic *Bifidobacterium breve* M-16V) and an amino acid–based formula (AAF) in infants aged 13 months or younger who have IgE-mediated CMA did not show any difference from AAF alone.^124^ However, stool samples collected in this study for analysis of fecal microbiota showed a higher percentage to bifidobacteria in AAF-plus-synbiotic group at both 6 and 12 months than in the AAF group.

Transplantation of healthy fecal microbiota or defined commensal bacterial taxa can potentially modify intestinal immune responses through restoration of gut immune regulatory checkpoints, mainly related to retinoic orphan receptor gamma T-positive (RORγt^+^) Treg cells, the epithelial barrier, and IgA response to gut commensals.^125^ Initial *in vitro* and animal studies have led to clinical trials evaluating the efficacy of fecal microbiota transplantation in FA (eg, a phase II study to evaluate the safety and tolerability of oral encapsulated fecal microbial transplantation therapy for patients with PA [NCT05695261] is ongoing). A recent SR showed that fecal microbiota transplantation is a promising strategy to prevent allergic symptoms, but further investigation is still required.^126^ Further studies are needed to evaluate which microorganism are best, which is the optimal dose, whether treatment needs to be continued, and whether the effects are maintained over the long term. Another approach being tested is the administration of antibiotics before or during peanut OIT (Table II).

### Allergen-specific vaccines

Peanut virus-like particles (VLPs) have shown, in a mouse model of PA, that the fusion vaccine using Ara h 2 is highly immunogenic and confers protection against local and systemic reactions to peanut. As the concentration of Ara h 2 is relatively low, the potential interaction and activation of MCs and basophils is reduced.^127^^,^^128^ Peanut VLPs are currently being evaluated in humans in a phase I study (Table II). *Prunus persica* 3 VLPs have also been evaluated in a mouse model with promising results.^129^

Peptide-based AIT is another approach that may minimize the risk of IgE-mediated reactions, as peptides may be too small to bind and cross-link IgE.^130^^,^^131^ PVX108, a mixture of short, synthetic peptides derived from peanut allergens administered intradermally has shown a favorable profile, with changes in allergen-specific T cells and skin prick test response persisting after dosing. Currently, a phase II study in children and adolescents with PA is ongoing (NCT05621317).

Additional approaches being studied in individuals with PA are as follows: ASP0892 (ARA-LAMP-vax), a single multivalent peanut (Ara h 1, Ara h 2, and Ara h 3) lysosomal-associated membrane protein DNA plasmid vaccine administered intradermally (NCT03755713), and nanoparticles coated with peanut protein (CNP-201) administered by intravenous infusion (NCT05250856). mRNA vaccines encoding allergens may be the next approach to be tried, given the success of this mode of delivery in the coronavirus disease 2019 (COVID-19) pandemic, which was worthy of the Nobel Prize in Physiology and Medicine 2023.

## FA prevention

Important research in FA development in early childhood over the past 15 to 20 years has motivated the transition from passive allergen avoidance to active management based on prompt interventions on eczema-affected skin and early introduction of allergenic foods. Maternal interventions during pregnancy and lactation have had more controversial results (Table III).

**Table III:** Current and ongoing research studies in FA prevention

Study name (NCT or ACTRN no.)	Study description	Estimated enrollment	Target age group	Main outcomes
Maternal interventions during pregnancy and lactation				
Can Vitamin D Supplementation in the First Year of Life Prevent Food Allergy in Infants? The VITALITY Trial: Parts 1 & 2 (the VITALITY Study; NCT02112734)	Phase 4, randomized, parallel assignment, quadruple masking, prevention study (∼6 years)	N = 2739	6-12 wk	Prevalence of challenge-proven FA at age 12 mo. Occurrence of definite FA or tolerance at age 6 y by combining data from an OFC, an SPT, and/or serum specific IgE test, and/or parent- or self-reported ingestion history, and reactions to the index food
The Role of *Bifidobacterium* Intervention in Food-Allergic Infants (NCT05965063)	Randomized, parallel assignment, open-label, treatment study with *Bifidobacterium* intervention (∼12 wk) Food allergies include CM, egg, soy, and wheat	N = 300	0-12 mo	Clinical symptoms of infants at the fourth wk by milk-related symptom score (0-33 [higher scores means worse outcomes])
Maternal Diet Rich in Eggs and Peanuts to Reduce Food Allergies: A Randomized Controlled Trial (the PrEggNut Study; ACTRN12618000937213)	Randomized, multicenter, parallel, 2-arm (1:1 allocation), single-blinded, controlled study of a diet rich in egg and peanut during pregnancy	N = 2136	Pregnant women at ≤wk 23 of gestation	Food challenge–proven IgE-mediated EA and/or PA in the infants at age 12 mo IgE-mediated EA and/or PA is defined as an allergic reaction to egg with sensitization to egg and/or an allergic reaction to peanut with sensitization to peanut. Sensitization is defined as ≥3-mm weal size based on an SPT. Infants with a positive SPT who do not proceed with the corresponding food challenge because of previous anaphylaxis or allergic reaction will be considered positive for IgE-mediated EA and/or PA; otherwise, the allergic reaction will be established according to an OFC
Skin barrier: Emollients, steroids and microbiome				
Moisturizer-Mediated Prevention of Symptoms of Atopic Dermatitis in Early Childhood (the MOPAD Study; NCT04398758)	Phase III, multicenter, randomized, non–treatment-controlled, investigator-blinded study of SanaCutan Basiscreme (∼12 mo)	N = 360	< 3 wk	Cumulative incidence of children with AD in the treatment group at age 6 mo is significantly lower than in the control group without predetermined treatment (*P* <.05)
Bathing Babies and Allergy (the BBA study; NCT03050658)	Prospective, observational cohort study of impact of a baby's first bath on his/her TEWL and skin microflora (∼2 y)	N = 144	≤72 h of life	Change in skin TEWL at 18-36 hours after the baby’s first bath in the hospital vs baseline
Seal, Stopping Eczema and Allergy Study (the SEAL; NCT03742414)	Phase II, randomized, controlled, parallel design, open-label clinical study of proactive sequential skin care, including the twice-daily use of a trilipid skin barrier cream (Epiceram) or moisturizer and proactive use of fluticasone propionate cream, against reactive AD therapy (∼3 y)	N = 875	1-12 wk	• Occurrence and severity of AD in early infancy • Per-participant cumulative no. of challenge-proven FAs at 3 y Food allergens included are egg, CM, peanut, sesame, fish, wheat, and 9 tree nuts
Allergic Disease Onset Prevention Study (the ADORED Study; NCT05003804)	Phase Ib and II, multicenter, randomized, double-blind, placebo-controlled study of STMC-103H (∼672 d [22 mo and 3 d]) STMC-103H is a combination of bacterial species that have been found to be depleted in the gut microbiota of infants who go on to develop allergic sensitization and allergic diseases in childhood	N = 264	0-14 d Neonate and infants subjects at risk for development of atopic disease	Parts A1 and A2: AEs, SAEs, and AESIs (at ≤d 56 of study) Part B: • AEs, SAEs, AESI, physical examination findings, and clinical safety laboratory test results (at ≤d 672 of study) • incidence of physician-diagnosed AD at d 336 Secondary outcomes: • Proportion of subjects who develop any atopic disease (AD, FA, allergic rhinitis/conjunctivitis, asthma), • Incidence of sensitization to food and aeroallergen and incidence of physician-diagnosed FA, allergic rhinitis/conjunctivitis, urticaria, and wheezing illnesses/asthma
Restoration of Microbiota in Neonates (RoMaNs Study; NCT03928431)	Randomized, single-center, parallel assignment, triple-masking, placebo-controlled study of exposure to the maternal vaginal and fecal microbiota directly after birth (∼2 y)	N = 300	Newborns of mothers aged 18-40 y	Cumulative incidence of IgE-associated allergic disease at age 2 y in infants delivered by cesarean section vs in nontreated infants delivered by cesarean section SPTs will be performed in infancy (at age 6 mo and age 12 mo) and childhood (at age 24 mo)
Early introduction and natural tolerance development				
Preterm Infants on Early Solid Foods (PIES-Project; NCT01809548)	Randomized, parallel assignment, prospective 2-arm intervention study (∼5 y)	N = 177	1 wk-3 mo	o Height difference at age 1 year, corrected for prematurity o Height will be measured under standardized conditions in cm at defined times during the first year of life until age 1 y, corrected for prematurity. Measurements will be done before, within, and after intervention to demonstrate the changes due to all intervention tools Secondary outcomes: - FX5 (specific IgE levels of food allergens at age 5 y) - SCORAD
The TreEat Study: Can Early Introduction of Tree Nuts Prevent Tree Nut Allergy in Infants with Peanut Allergy (the TreEat Study; NCT04801823)	Phase III, randomized, 2-arm, open-label, controlled study of a supervised hospital based multitree nut (almond, cashew, hazelnut, and walnut) OFC and then home introduction of the remaining tree nuts vs standard care (home introduction of all 8 tree nuts) in infants with PA to reduce the risk of developing tree nut allergy (∼18 mo)	N = 200	4-11 mo	Difference between the 2 treatment arms in the proportion of participants with clinically confirmed tree nut allergy at age 18 mo Tree nut allergy outcomes at 18 mo will be defined as - Having an allergy (has evidence of tree nut sensitization [SPT result ≥ 3 mm] and having had a reaction consistent with IgE-mediated FA *OR* positive formal OFC) - Tolerant of tree nuts (successfully tolerated 1 tsp of the tree nut per occasion at home on >3 occasions *OR* has had a negative formal OFC result) - Inconclusive (has an unknown outcome, as ingestion has not occurred and participant has declined OFC)

Details of the studies, including recruitment status, are as of October 2023 (https://clinicaltrials.gov/).

*ACTRN*, Australian New Zealand Clinical Trials Registry number; *AD*, atopic dermatitis; *AE*, adverse event; *AESI*, adverse event of special interest; *NCT*, National Clinical Trial; *SAE*, serious adverse events; *SCORAD*, Scoring Atopic Dermatitis; *SPT*, skin prick test; *TEWL*, transepidermal water loss.

### Maternal interventions during pregnancy and lactation

Healthy diet diversity during pregnancy and in the first year of life, which can result in changes in the gut microbiome, has been associated with lower odds of FA. Venter et al created the maternal diet index using data from the Healthy Start, a prebirth cohort of mother-offspring dyads.^132^^,^^133^ The index considered the consumption of vegetables, yogurt, fried potatoes, rice and grains, red meats, pure fruit juice, and cold cereals. Vegetables and yogurt were associated with the prevention of allergic disease. In adjusted models, a 1-unit increase in the maternal diet index was associated with a decreased risk of atopic diseases, except for FA.

An important intervention to study is the consumption of allergenic foods during pregnancy and whether this changes the FA risk in the offspring. As an example, the PrEggNut study is evaluating the effect that a maternal diet rich in eggs and peanuts has on egg and PA outcomes in their toddlers at 12 months of age.^134^ A recent mouse study suggests that application of *Bifidobacterium* *bifidum* TMC3115 during pregnancy shapes offspring gut microbiota, which may induce tolerance to allergens.^135^ Preliminary results suggest protective and anti-inflammatory properties of *Bifidobacterium infantis*^136^ and motivated a new trial assessing its effect on symptoms at the fourth week in infants with allergy to 1 of the following foods: cow's milk, egg, soya, and wheat (NCT05965063).

### Skin interventions in infancy

The dual allergen exposure hypothesis proposes that FA risk results from the balance between dose, timing, and route of exposure to food allergens in infancy, with high-dose exposure through the oral route being tolerogenic and low-dose exposure through the skin, particularly inflamed skin affected with eczema, being proallergenic.^137^ This hypothesis was supported by the LEAP study findings a few years ago^138^^,^^139^ and has motivated trials of skin interventions to prevent sensitization and development of FA. However, the findings have been somewhat surprising, with the BEEP study showing negative results for the preventative effects of emollients on FA risk and the PreventADALL study showing that emollients and early food introduction did not prevent eczema at age 12 months but did prevent FA at age 36 months.^140^, ^141^, ^142^ In the Enquiring about Tolerance (EAT) study, regular application of moisturizers to the skin of young infants was associated with increased transcutaneous sensitization and FA.^143^ Specifically, moisturization frequency showed a dose-response relationship with the development of FA in children with and without visible eczema at enrollment.^143^ Two possible explanations have emerged for these findings: either moisturizers facilitate the penetration of food allergens across the skin barrier and/or allergenic proteins are transferred to the babies’ skin by parents’ hands. A Cochrane review concluded that skin care interventions in infancy did not prevent eczema and may increase the risk of skin infection and FA.^144^

Given that eczema is a risk factor for FA, the question of whether early proactive treatment or reactive treatment of eczema is best for FA prevention has been addressed. For example, in the Prevention of Allergy via cutaneous Intervention (PACI) study, infants were randomized to proactively apply emollients and topical corticosteroids to both lesional and nonlesional skin (enhanced treatment) or to use topical corticosteroids on lesional skin reactively when needed (conventional treatment). The incidence of EA was significantly reduced with use of enhanced treatment (31.4%) versus conventional treatment (41.9%). However, the enhanced treatment lowered body weight and height; thus, lower potency topical corticosteroids or other topical therapies should be considered.^145^ Along a similar line, the Stopping Eczema and Allergy (SEAL) study aims to compare the effect of proactive sequential skin care or moisturizer with proactive use of fluticasone propionate cream to reduce the occurrence, exacerbation, and severity of atopic dermatitis, and therefore, to prevent FA (NCT03742414).

### Dietary interventions in infancy

A variety of prevention studies have examined early introduction of allergenic foods, in both the general population and high-risk infants. The main allergenic foods studied were peanut, egg, and CM. The landmark LEAP study showed the effectiveness of early peanut introduction in high-risk children aged 4 to 11 months, with those with severe eczema and/or EA considered high-risk individuals.^138^^,^^139^ The EAT study focused on the general population, and a significant reduction in PA and EA was seen only in the per-protocol analyses for children in the intervention arm.^146^ In a recent pooled analyses of the LEAP and EAT data, a significant reduction was seen with early introduction in all risk subgroups,^147^ highlighting the need to intervene at the level of the whole population, especially in countries in which peanut is an important food.^148^ The efficacy of the intervention increased with earlier age of introduction^147^^,^^148^ and was reduced if delayed until age 12 months.^148^ In a recent meta-analysis, early introduction of peanut from age 3 months to age 10 months was associated with a reduced risk of PA.^149^

Following the modification of the early introduction guidelines in Australia, there has been a preliminary indication of a reduction in food anaphylaxis admission rates in 1- to 14-year-olds. However, it seems that an increase in FA rate was seen in those younger than 1 year, which may be explained by earlier presentation of pre-existing FA with the introduction of allergenic solids in the first year of life.^150^ Specifically for PA, cross-sectional analyses did not reveal changes in prevalence of PA across the population.^151^ There are a variety of factors that could lead to this; they include not enough time for implementation of guidelines and follow-up of infants affected, no strict adherence to the appropriate amount and frequency of consumption, lifestyle choices, influence of cultural practices, and access to accurate information about weaning strategies. Many children with PA also avoid tree nuts. The TreEAT Study will evaluate whether early introduction of nuts can prevent allergy development in aged 4 to 11 months who have PA (NCT04801823).

The evidence that early introduction of CM can prevent CMA is conflicting.^149^ For instance, in the EAT and PreventADALL studies, no difference between the early introduction and standard introduction groups was found in the development of CMA.^141^^,^^143^^,^^146^ The ABC trial noted higher rates of sensitization to CM and CMA in those who had been receiving CM formula since their first day of life. However, these results should be interpreted with caution, as the time before discontinuation differed broadly among infants. In the SPADE study, in which infants were randomized to introduction of CM at age 1 month or avoidance during the first and second months of life, a reduction of CMA was observed in the group with daily ingestion of CM formula during the first and second months of life.^152^ Interestingly, in infants with exposure to CM in the first 3 days of life, CMA development was associated with CM formula discontinuation.^153^

Introduction of solid foods in the diet can have benefits other than exposure to the allergen via the tolerogenic gastrointestinal tract. In the EAT study, introduction of allergenic foods from the age of 3 months promoted a significant increase in gut microbiome diversity, specific microbes in the gut, and differential dynamics of maturation of the gut microbial communities versus in infants to whom solid foods were introduced only after age 6 months.^154^ A recent prospective study showed that infants with high diet diversity scores had lower gene expression of IL-4, IL5, IL-6, IL-8, and IL-13 and greater microbial diversity than observed in the group with lower diet diversity. The same study associated these changes with achieving oral tolerance and reducing the possibility of developing EA in high-risk infants.^155^

## Conclusions

We are living exciting times in FA research, with many promising fundamental and translational studies in the pipeline. The dissection of the immune mechanisms of FA and the identification of new targets for a definitive treatment should be a priority. A curative treatment is much needed, and the identification of specific targets in the aberrant allergic response are the key to unlock a true modification of this condition. An improved understanding of the epidemiologic and genetic changes in FA can help design specific interventions. It is hoped that ongoing clinical trials will lead us to more precise diagnostics and more active management of FA. Overall, such therapeutic interventions, and particularly preventive measures, can reduce the prevalence of FA and reduce its burden on patients’ lives and the society.

## Disclosure statement

Supported by the 10.13039/501100000265Medical Research Council (grants MR/M008517/1, MC/PC/18052, and MR/T032081/1 [to A.F.S.]); 10.13039/100006423Food Allergy Research and Education (to A.F.S.); the 10.13039/100014247Immune Tolerance Network/10.13039/100000060National Institute of Allergy and Infectious Diseases, National Institutes of Health (to A.F.S.); 10.13039/501100000362Asthma UK (grant AUK-BC-2015-01 [to A.F.S.]); the 10.13039/501100000268Biotechnology and Biological Sciences Research Council (to A.F.S.), the 10.13039/501100000833Rosetrees Trust (to A.F.S.) and the 10.13039/501100000272National Institute for Health Research through the 10.13039/100014461Biomedical Research Centre Award to Guy's and St. Thomas' NHS Foundation Trust (to A.F.S.) during the conduct of the study.

Disclosure of potential conflict of interest: A. F. Santos reports personal fees from 10.13039/100005180Thermo Scientific, Nutricia, Infomed, Novartis, Allergy Therapeutics, and Buhlmann, as well as research support from Buhlmann and 10.13039/100011033Thermo Fisher Scientific through a collaboration agreement with 10.13039/100009360King's College London. The rest of the authors declare that they have no relevant conflicts of interest.
